# Foodborne Infections and *Salmonella*: Current Primary Prevention Tools and Future Perspectives

**DOI:** 10.3390/vaccines13010029

**Published:** 2024-12-31

**Authors:** Antonella Zizza, Alessandra Fallucca, Marcello Guido, Vincenzo Restivo, Marco Roveta, Cecilia Trucchi

**Affiliations:** 1 Institute of Clinical Physiology, National Research Council, 73100 Lecce, Italy; antonella.zizza@cnr.it; 2 Department of Health Promotion, Mother and Child Care, Internal Medicine and Medical Specialities, University of Palermo, 90127 Palermo, Italy; alessandra.fallucca@unipa.it; 3 Laboratory of Hygiene, Department of Biological and Environmental Sciences and Technologies, University of Salento, 73100 Lecce, Italy; marcello.guido@unisalento.it; 4 School of Medicine, University Kore of Enna, 94100 Enna, Italy; vincenzo.restivo@unikore.it; 5 Food Hygiene and Nutrition Service, Local Health Unit 3, Department of Prevention, 16142 Genoa, Italy; marco.roveta@asl3.liguria.it

**Keywords:** *S. typhi*, *S. paratyphi*, non-typhoidal *Salmonella*, epidemiology, prevention, foodborne infections, vaccination

## Abstract

*Salmonella* is considered the major zoonotic and foodborne pathogen responsible for human infections. It includes the serovars causing typhoid fever (*S. typhi* and *S. paratyphi*) and the non-typhoidal salmonella (NTS) serovars (*S. enteritidis* and *S. typhimurium*), causing enteric infections known as “Salmonellosis”. NTS represents a major public health burden worldwide. The consumption of *S. enteritidis*-contaminated animal foods is the main source of this disease in humans, and eradicating bacteria from animals remains a challenge. NTS causes various clinical manifestations, depending on the quantity of bacteria present in the food and the immune status of the infected individual, ranging from localized, self-limiting gastroenteritis to more serious systemic infections. Salmonellosis prevention is based on hygienic and behavioral rules related to food handling that aim to reduce the risk of infection. However, no vaccine against NTS is available for human use. This aspect, in addition to the increase in multidrug-resistant strains and the high morbidity, mortality, and socioeconomic costs of NTS-related diseases, makes the development of new prevention and control strategies urgently needed. The success of the vaccines used to protect against *S. typhi* encouraged the development of NTS vaccine candidates, including live attenuated, subunit-based, and recombinant-protein-based vaccines. In this review, we discuss the epidemiological burden of Salmonellosis and its primary prevention, focusing on the current status and future perspectives of the vaccines against NTS.

## 1. Introduction

Ingestion of food or beverages contaminated by biological agents can cause foodborne infections.

The most common pathogens responsible are bacteria such as *Salmonella* spp., *Campylobacter jejuni*, *Shigella* spp., *Escherichia coli*, *Yersinia enterocolitica*, *Listeria monocytogenes*, *Vibrio* spp., and *Bacillus cereus*; viruses such as *Norovirus*, *Rotavirus*, *Hepatitis* A, and *Hepatitis* E virus; and parasites such as *Cyclospora cayetanensis*, *Toxoplasma gondii*, and *Trichinella spiralis* [1].

*Salmonella* is one of the most common pathogens responsible for sporadic or widespread gastrointestinal infections and poses a global public health threat. It was first isolated in 1884 by Theobald Smith from the intestines of pigs infected with swine fever in a veterinary laboratory in the United States [2]. Approximately 80% of human *Salmonella* infections occur as sporadic cases, often undiagnosed or underreported, rather than being associated with a known epidemic [3,4].

*Salmonella* is a genus of the *Enterobacteriaceae* family and is a Gram-negative, facultative anaerobic, rod-shaped bacterium, 2–5 μm long by 0.5–1.5 μm wide, and its genome varies from 4460 to 4857 kb depending on the serotype [5]. The nomenclature and classification of *Salmonella* are quite controversial and still evolving.

According to the terminology established by the Centers for Disease Control (CDC), the *Salmonella* genus currently comprises two species: *S. enterica*, which is the type species, and *S. bongori*. This classification is based on differences in the sequence of the 16S rRNA gene [6].

*S. enterica* is classified based on its genomic relatedness and biochemical properties [7] and includes six subspecies: *enterica* (I), *salamae* (II), *arizonae* (IIIa), *diarizonae* (IIIb), *houtenae* (IV), and *indica* (VI) [8].

*Salmonella* was classified by Kauffman and White based on the surface structures expressed on the bacterial lipopolysaccharides, flagella, and capsular polysaccharides [9].

The current classification is updated annually by the WHO Collaborating Centre for Reference and Research on Salmonella [10].

Currently, 22 “serovars” or “serotypes” belonging to *S. bongori* and more than 2600 serovars of the *S. enterica* species have been identified based on their antigenicity and ability to infect a wide range of hosts, of which over half (1586 serovars) belong to *S. enterica* subsp. *enterica* and are responsible for approximately 99% of all cases of *Salmonella* infections in humans and warm-blooded animals [11].

*S. bongori* and the other five subspecies of *S. enterica* are mainly isolated from cold-blooded animals and environmental sources; and they only occasionally infect humans [12,13].

Although the name “*S. enterica*” has been adopted by the CDC and WHO, specific serotypes of *Salmonella* are designated without the subspecies; for instance, *S. enterica* subsp. *enterica* serotype Typhi is abbreviated as *S.* ser. *typhi* or simply *S. typhi* [14].

From a clinical perspective, the serotypes of *S. enterica* subsp. *enterica* are classified based on their host specificity and the disease they can cause.

A small subset of serotypes, known as “specialists” can infect only a very narrow range of hosts. This group includes *S. typhi*, *S. paratyphi* A, *S. paratyphi* B, *S. paratyphi* C, and *S.* Sendai, collectively referred to as typhoid serovars. These serovars exclusively infect humans and higher primates, potentially leading to severe and life-threatening disseminated septicemic infection known as typhoid fever or enteric fever (EF) [15].

Non-typhoidal *Salmonella* (NTS) serovars are recognized as “generalists” due to their broad host spectrum, as they can infect various animal species, including humans, pigs, cattle, and poultry [16,17,18]. Most NTS infections result in self-limiting gastroenteritis, but in some cases, they can cause invasive non-typhoidal *Salmonella* (iNTS) disease. The likelihood of developing iNTS depends on factors such as the infecting serotype and species, the genetic background, and the immune status of the host. Examples of generalist serovars are *S. enteritidis*, *S. typhimurium*, monophasic *S. typhimurium*, *S. infantis*, and *S. derby*, which are the top five serovars in human surveillance data from the European Union, although their distribution varies in other WHO regions [19].

NTS serovars are classified into 67 serogroups based on the O antigen. Additionally, they can be further categorized into more than 2500 serovars based on the flagellar H antigen [5].

Moreover, there is a third group of serovars known as host-adapted serotypes that are typically associated with specific animal hosts and can occasionally cause invasive septicemic disease, resembling bacteremia, which occurs following infections with typhoidal serovars [20,21].

The objective of this review is to delve into the epidemiological aspects of salmonellosis and primary prevention strategies based on hygienic and behavioral practices related to food handling, with particular attention to the current status and prospects of vaccines against NTS.

## 2. Epidemiology

Despite significant advances in pathogen control and food cleaning strategies, infections by *S. enterica* subsp. *enterica* causes a high number of infections, hospitalizations, and deaths worldwide every year.

**Figure 1:**
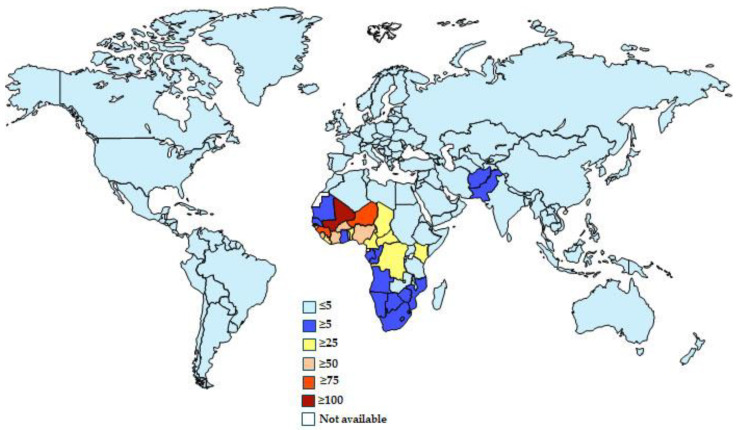
Estimated national invasive non-typhoidal *Salmonella* incidence (cases per 100,000 inhabitants) worldwide in 2024. From the Institute for Health Metrics and Evaluation [25].

**Figure 2:**
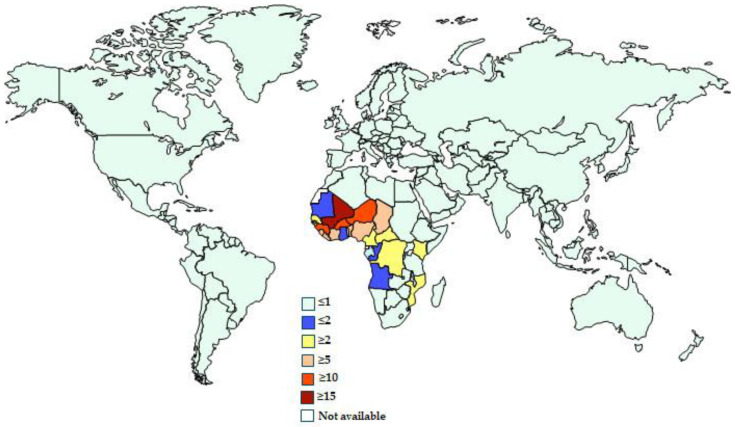
Deaths per 100,000 inhabitants due to invasive non-typhoidal *Salmonella* worldwide in 2024. From the Institute for Health Metrics and Evaluation [25].

The Global Burden of Disease Study (GBD) in 2019 estimated 9,237,224 million *S. typhi* cases [95% uncertainty interval (UI) = 5.94–14.13] (Figure 1), 110,029 deaths [95% UI = 52,810–1,891,205] (Figure 2), and 8.53 million disability-adjusted life years (DALYs) [95% UI = 3.86–13.92] [22].

The highest incidence was recorded in South-East Asia and Africa. In South-East Asia, the most affected countries were Pakistan (527 cases per 100,000 people), India (345 cases per 100,000 people), Bangladesh (304 cases per 100,000 people), Nepal (271 cases per 100,000 people), Papua New Guinea (263 cases per 100,000 people), and Indonesia (228 cases per 100,000 people). In Africa, the highest numbers of new cases were observed in Burkina Faso (355 cases per 100,000 people) and Kenya (251 cases per 100,000 people) [22].

The GBD estimated 3.79 million cases of paratyphoid fever [95% UI = 2.36–6.11], 23,337 deaths [95% UI = 9800–45,679], and 1.63 million DALYs [95% UI = 0.68–3.21] [22].

As can be seen from these data, infections caused by *S.* Typhi and Paratyphi occur more frequently in developing countries, while they are rather rare in America and Europe, with fewer than 10 cases per 100,000 people [23]. Most cases in these regions are linked to travelers returning from countries where these infections have a high incidence [24].

In 2019, the GBD estimated that iNTS disease, a leading cause of self-limiting diarrheal disease worldwide, would result in 593,877 cases [95% UI = 485,609–717,629], 79,045 deaths [95% UI = 43,012–124,206], and 6.11 million (3.32–9.71) global DALYs [Figure 1] [22].

The highest incidence was recorded in sub-Saharan Africa (43.2 cases per 100,000 person-years [95% UI = 34.8–53.5]) and in children under 5 years of age (163.9 cases per 100,000 person-years [95% UI = 119.0–219.0]).

The countries with the highest recorded incidence were Mali (110.2 cases per 100,000 person-years; 95% UI = 86.7–139.2), Nigeria (103.2 cases per 100,000 person-years; 95% UI = 84.4–124.9), Niger (80.3 cases per 100,000 person-years; 95% UI = 61.2–106.2), Burkina Faso (78.7 cases per 100,000 person-years; 95% UI = 60.1–100.9), Guinea (76.6 cases per 100,000 person-years; 95% UI = 59.9–100.7), and Sierra Leone (69.3 cases per 100,000 person-years; 95% UI = 54.1–89.5).

The mortality ratio was higher among children under 5 years of age (7.52 cases per 100,000 persons; 95% UI = 4.1–12.1) and in sub-Saharan Africa (6.26 [95% UI = 3.44–9.84]).

Countries with the highest recorded mortality were Mali (23.57 deaths per 100,000 [95% UI = 12.57–38.25]), Burkina Faso (16.91 deaths per 100,000 [95% UI = 8.86–28]), Niger (16.48 deaths per 100,000 [95% UI = 7.87–28.96]), Nigeria (16.38 deaths per 100,000 [95% UI = 9.2–25.31]), Guinea (13.89 deaths per 100,000 [95% UI = 7.3–23.37]), and Sierra Leone (12.52 deaths per 100,000 [95% UI = 6.38–21.26]) [Figure 2] [22].

In several regions of sub-Saharan Africa, a higher prevalence of iNTS disease has been observed during the rainy season, and this may be due to greater environmental exposure to the pathogen linked to fecal contamination of water and food or increased host susceptibility to invasive disease as a consequence of higher malaria transmission during this period of the year [25,26].

A systematic analysis of the burden of iNTS reports that few studies have been published on the incidence of NTS in Asia, where iNTS disease is less frequent, and there are no studies from Latin America. In contrast, in Europe and the United States, the incidence is less than 1 case per 100,000 person-years, and the mortality is less than 1 case per million inhabitants, with a percentage of cases between 10 and 24.9% attributable to HIV infection [27].

The most recent report from the European Food Safety Authority (EFSA) and the European Centre for Disease Prevention and Control (ECDC) estimated that over 350,000 cases of foodborne diseases occur annually in the European Union. However, the actual number is likely much higher [28]. Salmonellosis is the second most commonly reported foodborne gastrointestinal infection in humans and is a major cause of foodborne outbreaks in EU member and non-member states.

In 2022, there were 65,208 confirmed cases of salmonellosis reported, an increase from 60,169 cases in 2021. The notification rate in the EU remained steady at 15.3 cases per 100,000 inhabitants over these two years.

In particular, the highest notification rate in 2022 was reported in Czechia and Slovakia, with 71.9 and 67.5 cases per 100,000 inhabitants respectively. Italy is among the countries with the lowest percentages (5.6 cases per 100,000 inhabitants) together with Bulgaria, Greece, Latvia, Portugal, and Romania, which report less than 6.1 cases per 100,000 inhabitants.

In the EU, 62.3% of salmonellosis cases were acquired, a decrease of 10.5% compared to 2021. In addition, 5.0% of cases were linked to travel outside the EU, while 32.7% had unknown origin. The ratio of domestic versus travel-associated cases varied significantly between countries. Of the 4135 travel-associated cases, 77.8% involved travel outside the EU. The most commonly reported countries of origin for these cases were Turkey (24.8%), Egypt (11.2%), Morocco (7.0%), and Thailand (5.3%). Spain and Italy are the most frequently reported travel destinations in the EU for these infections.

A seasonal trend was observed for confirmed cases of salmonellosis in the EU over 2013–2022, with the highest number of cases during the summer season.

The hospitalized rate in 2022 was 38.9%, slightly higher than in 2021, with 11,287 cases hospitalized reported by seventeen member states (MSs) and 81 fatal cases reported by nine countries, with the highest rate of fatal cases (7.2%) for blood infection.

The main *Salmonella* serovars responsible for human infections were *S*. *enteritidis* (67.3%) and *S. typhimurium* (13.1%), monophasic *S. typhimurium* (4.3%), *S. infantis* (2.3%), and *S. derby* (0.89%).

In 2022, among “ready-to-eat” products, 0.16% of 99,341 samples tested positive for *Salmonella*, as reported by 25 MSs. The highest levels of contamination were found in “meat and chicken meat products” (1.4%; N = 584) and “spices and herbs” (1.1%; N = 1309), while in “not-ready to eat” products 2.1% of 521,917 samples reported by 28 MSs were positive for *Salmonella*, with the highest levels in “chicken meat and meat products” (5.1%; N = 99,022) and turkey (3.3%; N = 13,867).

In 2022, *Salmonella* continued to be the most frequently reported cause of foodborne outbreaks. A total of 1014 foodborne outbreaks occurred in the EU, with an increase of almost 24% compared with 2021 (773 outbreaks) reported by 27 EU MSs, the United Kingdom (Northern Ireland), and 11 non-MSs, causing 6632 cases of illness, 1406 hospitalizations, and eight deaths. *S. typhi* was responsible for five epidemics, 84 cases, and 20 hospitalizations.

Foods can also be contaminated by food handlers, who are responsible for preparing and serving it. They can significantly contribute to the spread of foodborne infections [29,30].

Many studies have been conducted in countries with poor sanitary conditions to investigate the prevalence of intestinal parasitic and enteric bacterial infections among food handlers.

The risk of *Salmonella* infection among food handlers varies significantly across countries and regions, influenced by factors such as climate, geography, and socioeconomic development. In developing countries, studies have found that the prevalence of infection among food handlers ranges from 3.1% to 8.2% [31,32,33,34].

However, there is limited research available regarding the prevalence of Salmonellosis among food handlers in developed countries.

### Surveillance and Monitoring of Salmonella in the EU

In all EU MSs, the notification of Salmonellosis is mandatory, with the exception of Belgium, France, and the Netherlands, where it is voluntary. In 2022, the surveillance system for Salmonellosis achieved national coverage in most countries. However, Belgium, the Netherlands, and Spain had estimated coverage levels of 85%, 64%, and 73% respectively.

Notification rates for human salmonellosis varied significantly among different MSs. These variations are influenced by factors such as the quality, coverage, and severity of disease as well as surveillance systems, sampling, and testing practices. For instance, the percentage of hospitalization varied between 22 and 100% across different countries. Countries with lower notification rates had a higher percentage of hospitalization, suggesting that the surveillance system in these countries mainly focused on the most severe cases [19].

Control and prevention measures for *Salmonella* in animals necessitate the development of a biosecurity system throughout the food production and processing chain [35,36].

In Europe, effective control and prevention measures for *Salmonella* transmission in the agri-food chain encompassing primary production, food processing, post-processing, and the final product have been established through Directive 2003/99/EC and Regulation (EC) No. 2160/2003 [37].

Additionally, the EU has implemented a Zoonoses Monitoring System coordinated by the European Food Safety Authority (EFSA) and the European Centre for Disease Prevention and Control (ECDC) [38].

## 3. Route of Transmission and Pathogenesis

### 3.1. Transmission

*Salmonella* infects hosts via the fecal–oral route, through the ingestion of contaminated foods or drinks, or by direct contact through the manipulation of objects or animals in which the microorganisms are present [17].

Different *Salmonella* serotypes demonstrate host predilection and are usually associated with a specific host or animal reservoir representing different vehicles of the infection [34].

Typhoid serotypes are highly adapted to humans, and reservoirs of infection exist only among humans and some primates [39]. The transmission of typhoid fever depends on direct contact with the feces of infected individuals. The risk is higher in areas with poor sanitation and a lack of or no access to drinking water [40].

The transmission of typhoid fever is also associated with environmental factors such as the presence of uncovered sewers and contaminated water basins. Therefore, contaminated water constitutes an important environmental route for the spread of *S. typhi* [41,42].

Generalist serotypes [NTS] are capable of causing infections in both humans and animals; furthermore, unlike typhoid fever which is especially common in developing countries, NTS salmonellosis is widespread throughout the world [17].

The primary reservoir of NTS is the intestinal tract of various domestic and wild animals [43]. Some NTS serovars show a clear predilection for the animal reservoir, such as *S. dublin* and *S. choleraesuis*, which prefer cattle and pigs, respectively, as the specific hosts [44].

Contaminated food of animal origin, together with a variety of derived food products, represents one of the most common vehicles for the spread of NTS infection in humans. These NTS serotypes are mainly transmitted by foods such as contaminated beef, pork, poultry, milk, and eggs, although outbreaks of infection have also been documented transmitted via seafood, fruit, and vegetables, which can act as vehicles [45,46,47]. Food contamination can occur during all stages of food processing: slaughtering of meat, washing of meat with contaminated water, storage and refrigeration at inappropriate temperatures, incomplete cooking, and production of the finished or ready-to-use food [48,49].

Contamination of eggs has been identified as a major cause of foodborne *Salmonella* [50]. Contamination of eggs by *Salmonella* can occur directly during the formation of the egg in the reproductive tract of the hens or indirectly or after the laying of the egg due to *Salmonella* contamination that penetrates from the outside through the shell membrane [50,51]. Indirect contamination is often related to the food processing and handling process in which vehicles of infection can also be surfaces and tools if handled by infected personnel or in circumstances where hygiene and health standards are lacking [51].

The transmission of salmonellosis is particularly high in closed community settings with high crowding rates and with promiscuity characterized by the use of shared toilets, consumption of common meals prepared by the same kitchen, access to the same places, and contact with shared tools. Many outbreaks of salmonellosis are documented in settings with the characteristics of closed communities, such as kindergartens, early childhood schools, and hospitals [52,53].

A concomitant factor is the state of susceptibility of the host populations of such communities. The pediatric age group is characterized by immature immune defense, reduced macrophage activity, and deficient local immunity of secretory IgA [54,55]. Similarly, in advanced age, the immune system undergoes a paraphysiological senescence phenomenon that results in a decrease in immune defense. Patients affected by chronic conditions such as metabolic diseases, gastrointestinal diseases characterized by digestive distress, gastrointestinal neoplasms, and immunological suppression from pharmacological or radiation treatment appear more likely to be candidates for salmonellosis [55].

### 3.2. Pathogenesis

*Salmonella* enters the host through the gastrointestinal tract and translocates into deeper tissues through a mechanism of rearrangement of the cell membranes of the enterocytes, which is part of the internalization process [56]. Infection begins when microorganisms cross the epithelial surface of the small intestine, the primary site of *Salmonella* infection. In detail, these bacteria interact preferentially with Peyer’s patches, lymphoid tissue associated with the intestine [57]. This is followed by the degeneration of the microvilli and, subsequently, the development of vacuoles containing the bacterium by cytoplasmic projections of the enterocytes. Over time, the apical surface of enterocytes regenerates, apparently returning to normal [57].

Having reached the lymphoid tissue of Peyer’s patches, the microorganisms disseminate through the mesenteric lymph nodes and reach the lymph nodes of the spleen and liver. The extension of the infection occurs because *Salmonella* can survive and replicate in macrophages, a privileged environment for evading the adaptive immune response [58,59].

Furthermore, an important pathogenicity mechanism of *Salmonella* is represented by interference with the activity of dendritic cells, which prevents recognition by the host’s adaptive immunity [60].

Many host risk factors can predispose to *Salmonella* infection. First of all, the dose of ingested microorganisms can be correlated with the development of the disease. The symptoms of NTS infection depend on the number of bacteria ingested. Some authors suggest that the number of bacteria that must be introduced for a healthy human host to develop a symptomatic disease varies from 10^6^ to 10^8^ organisms. In immunocompromised infants and children, however, the disease can be caused by the ingestion of even a minimal dose of bacteria [54]. Other risk factors include gastric hypochlorhydria with a reduction in the first defensive barrier of the stomach, a characteristic of infants who, due to the rapid emptying of the stomach, have reduced gastric acidity, and a decrease in humoral and cellular immunity, a characteristic of older people and immunocompromised subjects [54,61].

## 4. Clinical Manifestations, Diagnosis, and Treatment of Non-Typhoidal *Salmonella*

### 4.1. Clinical Manifestations

*Salmonella* infections can assume a wide range of disease severity, varying from local carriage states, gastroenteritis, and diarrhea to bacteremia and extraintestinal infections [62]. In particular, the two serovars of the most pathogenic species *S. enterica* differ in terms of involved hosts and clinical presentation [62]. The typhoidal serovars *S*. *typhi* and *paratyphi* are restricted to humans and cause enteric fever [63], while the non-typhoidal serovars (NTS) *S*. *typhimurium* and *S*. *enteritidis* have a wide range of hosts and usually cause acute gastroenteritis that is mild and self-limiting [64,65].

However, certain strains of NTS termed invasive NTS (iNTS) have been proven to lead to fever and bacteremia, often in the absence of diarrhea [66]. Infants and young children, older individuals, immunocompromised or malnourished subjects, and those with recent malaria or HIV infection are at risk of developing iNTS [65,67,68,69,70,71].

Multiple body sites can be involved in the spread of NTS through the blood. Many of these complications are life-threatening [68,72] if untreated or improperly treated. A recent meta-analysis by Marchello CS et al. estimated that about 15% of patients affected by iNTS die [73].

The most serious short-term complications include endovascular and central nervous system infections. Endocarditis; musculoskeletal infections; visceral infections involving the spleen, liver, heart, lungs, or pleura; and urinary tract infections are other possible infection sites [74].

NTS meningitis affects infants and immunocompromised subjects. It is associated with significant morbidity and long-lasting neurological sequelae, potentially causing serious developmental delays and motor impairments (e.g., seizures, abscesses, hydrocephalus, and subdural empyema) [75]. Further, the case fatality is about 52% in Africa [62].

The vascular complications occur more frequently in patients affected by atherosclerosis and subjects previously subjected to vascular interventions [76].

Endocarditis occurs in 1–5% of the bacteremia and affects above all immunocompromised subjects and patients with renal failure and prosthetic valves [74]. This infection is characterized by a destructive course and a mortality rate above 45% [77]. Unified diagnostic criteria are lacking, making the diagnosis critical [78].

Other severe although rare complications include mycotic aneurysms, which consist of localized arterial wall dilatations caused by vessel wall damage. In comparison with atherosclerotic aneurysms, mycotic aneurysms are linked to a higher rupture incidence and mortality rate [79].

Pleuro-pulmonary manifestation consisting of pulmonary and pericardial abscesses has been observed in patients with pre-existing lung conditions [80].

Osteomyelitis is often linked to contact with pet reptiles and occurs more frequently in children with hemoglobinopathies than in adults [81]. The epiphysis of long bones and the axial skeleton is typically affected in prepubescent children and in adults, respectively [82]. Although rare, spondylodiscitis can occur in healthy and immunocompetent children [83].

The urinary tract can also be affected by *Salmonella* infections, in particular, in patients with diabetes, immunodeficiency, and genitourinary tract abnormalities (e.g., nephrolithiasis, chronic pyelonephritis, and urethrorectal or retrovesical fistulas). Of note, urinary tract infections can also occur in immunocompetent and healthy subjects [84].

Furthermore, NTS can persist in the host and lead to chronic diseases such as autoimmune disorders (e.g., reactive arthritis and inflammatory bowel disease) and neoplasms, such as colorectal and gallbladder carcinoma [85].

### 4.2. Diagnosis

In order to distinguish clinical symptoms of acute gastroenteritis associated with *Salmonella* infections from other enteric bacterial pathogens, the isolation of the bacterium from stool samples is still required [86]. The diagnosis of iNTS, instead, is based on the isolations of the pathogen from other systemic sites (e.g., the blood, lymph nodes, and bone marrow) [86]. Similarly, the diagnosis of enteric fever is conducted through the isolation of typhoidal serovars of *Salmonella* from the blood, bone marrow, urine, other sterile sites, or stool [86]. Following the identification of the species of *S. enterica*, the further typing of the serovar is usually conducted by central reference laboratories usually for surveillance, epidemiological studies, and outbreak investigations [86].

The serological diagnosis of enteric typhoid fever was carried out by the Widal tube agglutination test, but its efficacy has proven to be controversial. Thus, its use is now limited to the regions without advanced laboratory infrastructures [87]. Enteric fever point-of-care diagnostic tests, which test IgM and/or IgG antibodies against an outer membrane protein antigen or *S*. *typhi* LPS antigen, are currently available [88,89]. However, the moderate sensitivity and specificity of these tests for enteric fever diagnosis do not allow their substitution with blood culture [89,90]. Thus, typhoid fever is still laboratory tested by PCR or culture. In particular, molecular approaches have been clinically validated for the diagnosis of gastrointestinal NTS infection [91], iNTS [92], and TS infection in patients with enteric fever [93]. Further, PCR-based multiplex detection panels able to quickly identify *Salmonella* in stool samples have been developed [65,94,95,96]. Nevertheless, culture is still necessary for serovar classification and susceptibility testing [86]. The replacement of culture method with molecular tests could be evaluated when a better level of experience and a higher volume of analysis is obtained [97].

Of note, MALDI-TOF MS-based approaches are soon expected to become more relevant in *Salmonella* identification and typing [86].

Finally, the whole-genome sequencing (WGS) of bacterial genomes is the method of choice for *Salmonella* characterization and subtyping for epidemiological studies and to investigate persistent and recurrent infections [98,99,100,101,102,103].

### 4.3. Treatment

Self-limiting gastroenteritis and diarrhea caused by NTS are usually self-limiting within 3–4 days and do not require any such treatment [62]. Invasive NTS, instead, is frequently life-threatening, mostly for children, the elderly, and immunocompromised subjects [62]. Thus, the treatment of iNTS includes targeted antimicrobial therapy, intensive care, and surveillance [104]. In particular, the “Access group” of antimicrobials (first line) includes ampicillin, sulphamethoxazole/trimethoprim, and chloramphenicol, and the “Watch group” (second line) includes the third-generation cephalosporins, fluoroquinolones, and, in some cases, azithromycin [105,106].

A specific antimicrobial treatment based on the sites of infection is required for extraintestinal infections [62]. In particular, the therapy for bloodstream infections lasts 7–14 days, while focal infections (e.g., meningitis, osteomyelitis, and endocarditis) require 4–6 weeks of antibiotics [105].

Antibiotic treatment is not recommended for intestinal eradication of *Salmonella* in food handlers due to its poor efficacy and because it can lead to prolonged excretion of the organism and the development of antibiotic resistance [107,108].

Of note, an increase in antimicrobial resistance of NTS has been observed from the 1990s to the 21st century by surveillance data. This observation makes the therapy for Salmonellosis particularly critical [62,109].

The management of iNTS requires good sanitization, appropriate testing, intensive care, surveillance, and monitoring of the antimicrobial resistance. This allows the antimicrobial therapy to be prescribed consistently with the local epidemiologic burden and the antimicrobial resistance profile of the isolates [62].

## 5. Prevention: Hygiene Measures, Available and Future Vaccines, and Immunization Strategies

### 5.1. Sanitation Measures

Multidrug resistance of several *Salmonella* serotypes, especially iNTS, hinders the treatment and control of the disease. Prevention could effectively limit the spread of salmonellosis and reduce the disease burden. However, animals from breeding or destined for the food industry are still the main reservoirs of iNTS, and the primary focus of preventive practices has shifted to the hygienic–sanitary measures to be adopted throughout the entire food processing chain [110]. To reduce the incidence of food zoonoses, such as salmonellosis, preventive measures must be adopted during the production, processing, handling, and distribution of food of animal origin. These measures must be complemented by surveillance programs for diseases transmitted between farm animals and control of emerging outbreaks within the farm [111].

The complexity of the food industry system highlights the importance of a “One Health” approach, according to which human, animal, and environmental health cannot be addressed as distinct and separate issues. The multi-sectoral and multidisciplinary “One Health” approach responds to the need for clean water and air and safe and uncontaminated food, effectively addressing the threats to public health posed by food zoonoses [19].

A control strategy adopted in many farms in the most developed countries is livestock vaccination. Numerous pieces of evidence in the literature have shown the beneficial effect of vaccination on breeding animals. Regardless of the type of vaccine used, (live vs. attenuated) and the target strain of *Salmonella* (based on the geographical areas considered), a reduction in animal colonization has been documented by most studies which translates into a health benefit, the consumption of uncontaminated food from uninfected animals [112,113,114].

Regarding the field of food safety, in Europe, the “Hygiene Package” has standardized hygiene practices and related controls since 2000 to guarantee a high level of protection and safety in the food sector within the European community, ensuring the placing on the market of controlled, safe, and healthy foods [115].

Furthermore, with the implementation of procedures based on Hazard Analysis and Critical Control Points (HACCP) and with the identification and management of risks, all food handler workers involved in any level of the food production process have been made responsible [116].

The sanitization of all tools and machinery used for the production of food; washing of hands before, during, and after the preparation of food; refrigeration and blast chilling at adequate temperatures; cooking of food especially of animal origin; separation of raw foods from cooked foods during storage; transport via suitable containers; and constant training of food sector workers represent the key points of the health regulations adopted in Europe to guarantee safety in the food sector [117,118].

In developing countries, however, the WASH strategies launched in 1990 as an objective of sustainable development have over time guaranteed greater availability of clean water for the population and greater access to sanitation services [115,119]. Although WASH strategies represent a pillar for the control of salmonellosis, as well as for other infectious diarrheal diseases, they require long-term economic investments, continuous financing, and constant political commitment, which hinder developing countries [120].

Today, the countries with the greatest disease burden from *Salmonella* spp. aim for a comprehensive approach that also includes immunization, combining short-term prevention measures with more long-term solutions such as vaccination.

### 5.2. Available and Future Vaccines

Currently, two vaccines against *Salmonella* have been authorized and made available worldwide: the oral live attenuated Ty21a vaccine and the Vi-CPS polysaccharide vaccine containing the purified “Vi” antigen to be administered intramuscularly [121,122].

Neither vaccine has been implemented in national pediatric vaccination schedules due to poor immunogenicity. Several studies have shown a vaccine efficacy of around 50–70%, which does not persist in the long term [123].

The ability of *Salmonella* to survive within intracellular niches in macrophages represents an obstacle to the development of more effective vaccines. Both humoral immunity and cell-mediated immunity are necessary for the control and elimination of *Salmonella*. Circulating antibodies are necessary to counteract/neutralize bacteria when they are in an extracellular environment, but cell-mediated immunity is essential for the elimination of persistent infection within phagocytes [124].

Studies conducted on subjects suffering from immunodeficiencies and with congenital immune deficiencies support the importance of cell-mediated immunity to confer persistent and long-lasting protection [124].

Available vaccines induce the antibody immune response but do not stimulate T cells. The time needed for circulating antibodies to kill *Salmonella* is sufficient for the bacteria to take refuge in intracellular niches inside the macrophages, where they cannot be reached by antibodies [125].

In addition to evaluating how to activate the immune response to confer effective and long-lasting protection, it is important to understand which serotypes should be targeted by vaccines.

Both authorized vaccines are targeted exclusively against *S. typhy*. To date, there are no vaccines authorized by international health institutions that offer protection against other highly widespread serotypes responsible for invasive forms of disease [122].

Subunit vaccines based on antigens specific to groups of serotypes could guarantee broader immune coverage. The high costs associated with the development of these vaccines hinder/threaten their use, especially in developing countries where there are higher numbers of deaths and a more severe disease burden caused by *Salmonella* [126]. However, the evolving epidemiology typical of *Salmonella* serotypes spreading also represents an obstacle for serotype-specific vaccines, as the prevalence of the serotypes in a geographic area may change during the time necessary for the development of the specific vaccine [110].

The real challenge today is represented by the development of a polyvalent vaccine that can confer long-term protection against the main *Salmonella* serotypes responsible for serious enteritis and invasive diseases throughout the world: *S. typhimurium*, *S. enteritidis*, *S. typhi*, and *S. paratyphi*.

A bivalent vaccine against enteric fever is being developed in Asian territories [127], while a trivalent vaccine against NTS and *S. typhi* strains is being tested in Africa [128].

In Vietnam, a Vi polysaccharide vaccine conjugated to *Pseudomonas aeruginosa* exotoxin A (Vi-rEPA) and, in India, a Vi-tetanus toxoid conjugated typhoid vaccine (PedaTyph; in India) have both been prequalified by the WHO. These vaccines aim to address the significant burden of typhoid fever, particularly in regions where the disease is endemic [129].

Laboratory studies show that the combination of polysaccharide components, such as Vi antigen and O2, O4, and O9 antigens, with a carrier protein for activating the cellular response, such as diphtheria toxin DT, could lead to an effective quadrivalent vaccine useful for combating the four most widespread invasive serotypes [130,131]. In particular, a glycoconjugate vaccine, containing high-molecular-weight complexes of *Salmonella* Typhimurium O-specific polysaccharide (OSP) and recombinant T2544 that elicits protection against *S*. *typhi*, *S*. *paratyphi*, *S*. *typhimurium* and cross-protection against *S*. *enteritidis* in mice is under development. OSP-rT2544 could be a broad-spectrum candidate subunit vaccine against human infection due to typhoidal and non-typhoidal *Salmonella* serovars [132].

The implementation of a quadrivalent combined vaccine to be used without distinction in all geographical areas regardless of the specific epidemiology of the individual serotypes per area could be the most advantageous and also the most convenient solution [106].

As regards specific vaccines against NTS, recent evidence suggests that they have to be able to induce antibodies to multiple targets and that they need carriers as protein antigen components to induce effective humoral and cell mediated responses [133]. In particular, capsular polysaccharides (CPSs) (colonic acid, lipopolysaccharides (LPSs), and O-Ag capsules) are polymers expressed on the NTS surface that induce host immune response and are potential targets of vaccine development [134,135,136,137].

*Salmonella* O-Ag capsules have been used as targets for protective immunity against NTS in clinical studies conducted in healthy adults and children in the United States, eliciting serum bactericidal activity [138]. Nevertheless, unconjugated *Salmonella* O-Ag has showed limited immunogenicity; thus, OSP-based vaccines have been implemented by chemical conjugation to carrier proteins (e.g., TT, DT, CRM197, and FliC) to enhance the immune response [139,140,141]. Promising results in mouse experiments have been demonstrated, but they have been shown to be antigenically unique, making it necessary to combine different monovalent formulations to obtain a wide vaccine coverage against multiple serovars [131,142].

Interesting vaccine platforms are outer membrane vesicles (OMVs) produced by Gram-negative bacteria [143]. They can be used to present NTS cell surface protein antigens and lipopolysaccharides (LPSs) in their precise conformation and thus to elicit an effective immune response without the adverse risks of live attenuated vaccines [143]. Promising OMV-derived vaccines are based on genetically modified bacteria, which exhibit hyper-blebbing of the outer membranes, resulting in the development of Generalised Modules for Membrane Antigens (GMMA). In particular, an aluminum hydroxide-formulated GMMA vaccine from modified *S*. *typhimurium* and *S*. *enteritidis* strains administered intramuscularly is obtaining promising pre-clinical results [144]. Furthermore, phase 1 studies in healthy adults have been started [133].

An OMV-based bivalent immunogen was formulated as a vaccine candidate to elicit broad-spectrum protective immunity against *S*. *typhimurium* and *S*. *enteritidis*. The authors concluded that this vaccine could generate broad-spectrum immunity against prevalent iNTS mediated gastroenteritis [145].

Furthermore, a trivalent OMV-based vaccine candidate against *Campylobacter jejuni*, *S. typhimurium*, and *S. enteritidis* has been developed and used for intraperitoneal immunization of adult BALB/c mice, showing promising results [146].

As regards oral live attenuated vaccines, phase 1 studies were conducted to evaluate the effectiveness of CVD 1902 vaccine against *S*. *paratyphi* A, and preclinical studies investigated CVD 1921 and CVD 1941 vaccines against *S*. *typhimurium* [127].

Further, three live *S*. *typhimurium* vaccine strains (CVD 1921, CVD 1921 ∆htrA, and CVD 1926) have been assessed by Sears KT et al. in infant BALB/c mice to predict how they would perform following peroral immunization of infants. When administered in infant mice by intranasal administration, all the three vaccine strains were immunogenic, but CVD 1921 was the most protective [147].

Finally, subunit vaccines against the NTS strains have been also investigated, and to-date they have showed a modest efficacy [148,149].

### 5.3. Immunization Strategies

The WHO recommends the strategic use of available typhoid vaccines as part of efforts to control typhoid fever [131]. Introducing typhoid vaccines is a priority for countries with a high burden of typhoid disease or that have strains of *S.* Typhi that are resistant to antibiotics. Each country should select its vaccination strategy based on factors such as disease burden, risk factors for transmission, surveillance system, cost-effectiveness, affordability, and operational feasibility. The WHO also advises that vaccination should be implemented in response to confirmed outbreaks of typhoid fever. Furthermore, consideration should be given in humanitarian emergencies depending on the risk assessment, along with providing access to safe water and promoting improved sanitation and hygiene practices, especially among food handlers. Regarding the immunization of special populations, the WHO recommends typhoid vaccination of travelers from non-endemic to endemic areas, health-care workers, pregnant women, and HIV-infected individuals and those with other immunocompromising conditions. Of interest, professional food handlers in areas with the presence of typhoid are recognized as a high-risk group for acquiring or transmitting *S. typhi* infection.

Targeting infants and young children living in sub-Saharan Africa should be the priority for future vaccines against invasive non-typhoidal salmonella, given the high rates of incidence and significant disease burden faced by this vulnerable population. Outside of sub-Saharan Africa, vaccination efforts should focus on at-risk groups. In particular, invasive non-typhoidal *Salmonella* disease is common among immunocompromised individuals, including those with certain primary immunodeficiencies (such as Mendelian Susceptibility to Mycobacterial Disease, Chronic Granulomatous Disease, and Sickle Cell Disease) as well as HIV-infected subjects [105,150,151,152,153,154].

Further, food handlers could be evaluated as immunization targets because of their potential roles as reservoirs for NTS strains associated with invasive disease, as well as other adults operating in at-risk settings such as the food industry.

## 6. Conclusions

NTS represents a major public health concern, as it is responsible for high morbidity and mortality worldwide, especially in developing countries; thus, implementation of effective prevention strategies is required [155]. Effective prevention of Salmonellosis relies largely on minimizing food contamination, as the consumption of tainted food remains the primary route of transmission [156].

In addition to these “short-term” measures, vaccination represents a “long-term” approach to controlling the spread of the disease.

However, to date, the economic burden of vaccine development and the substantial continuous variability in *Salmonella* serotypes pose a significant challenge to *Salmonella* vaccine prevention strategies, and no vaccines are yet available against serovars other than *S. typhi*, thus including NTS [110,126,157].

Further studies are necessary to develop vaccines covering a broader spectrum of serotypes and capable of protecting against the other main *Salmonella* serotypes, including nontyphoidal *Salmonella* serovars.

This advancement could broaden prevention possibilities and policies, extending vaccination to encompass additional groups such as food handlers, vulnerable patients prone to severe clinical manifestations, or people living in endemic countries.

## Data Availability

No new data were created or analyzed in this study. Data sharing is not applicable to this article.
